# Unveiling the Cytotoxic and NO Inhibitory Potential of *Heliotropium dolosum* Extracts from Türkiye: A First Insight Into Its Phenolic Profile

**DOI:** 10.1007/s11130-025-01313-y

**Published:** 2025-03-06

**Authors:** Cennet Özay

**Affiliations:** Department of Basic Pharmaceutical Sciences, Faculty of Pharmacy, Izmir Katip Celebi University, Izmir, 35620 Türkiye

**Keywords:** Phenolic compounds, Antioxidant activity, HPLC assay, Cytotoxicity, Nitric oxide

## Abstract

*Heliotropium dolosum* (Boraginaceae) is traditionally used for its choleretic, antipyretic, and cicatrizing properties. Despite some reports on its traditional uses, this species’ cytotoxic and nitric oxide (NO) inhibitory potential and its polyphenolic compounds have not been previously investigated. This study aims to evaluate the antioxidant, cytotoxic, and NO inhibitory activities of *H. dolosum* extracts and to identify polyphenolic compounds that could be contributing to these effects. The antioxidant activity of *H. dolosum* extracts (ethanol, methanol, chloroform, water) was assessed using six in vitro models. Total phenolics, flavonoids, and saponins were quantified via colorimetric assays, while phenolic compounds were identified using HPLC. Cytotoxicity was evaluated through brine shrimp lethality and cancer cell assays, and NO inhibitory activity was measured in LPS-induced cancer cells. The methanol extract displayed the highest antioxidant activity, with major phenolic compounds identified as caffeic acid, 2,5-dihydroxybenzoic acid, and ellagic acid with 12.307, 5.710 and 5.061 mg/g, respectively. It showed notable cytotoxicity (LC₅₀ = 18.1 µg/mL) and concentration-dependent effects on cancer cells. The highest NO inhibition was observed in HCC78 cells (15.56 µM nitrite). *H. dolosum* demonstrates potential as a wild plant source of bioactive compounds with antioxidant, cytotoxic, and NO inhibitory properties, highlighting its promise for functional food development and nutraceutical applications.

**Supplementary Information:**

The online version contains supplementary material available at 10.1007/s11130-025-01313-y.

## Introduction

Centuries of traditional plant knowledge, preserved through ethnobotanical studies, hold immense significance for understanding and conserving the uses of native flora [1]. Modern research underscores the urgent need to investigate medicinal plants, driven by the burgeoning demand for bioactive compounds in the food, pharmaceutical, cosmetic, and nutraceutical sectors [2].

*Heliotropium* L., a key Boraginaceae genus, comprises 270–330 species worldwide, including 16 species in Türkiye, four of which are endemic [3]. Known for their rich phytochemical diversity, *Heliotropium* species harbor compounds such as pyrrolizidine alkaloids, terpenoids, phenolics, and quinones, renowned for their potent antioxidant and antimicrobial properties [4]. These antioxidants mitigate oxidative stress caused by reactive oxygen species (ROS), contributing to conditions like cancer, aging, rheumatoid arthritis, and arteriosclerosis [5].

Nitric oxide (NO) plays a vital role in various physiological processes; however, its excessive production can exacerbate tumor proliferation, highlighting the importance of identifying plant-derived inhibitors of NO synthesis for advancing cancer therapies [6]. Among plant-based bioactive compounds, phenolic acids such as caffeic acid and ellagic acid are particularly noteworthy for their dual role in exhibiting cytotoxic and anti-inflammatory properties. These compounds have been shown to induce apoptosis, suppress cancer cell proliferation, and inhibit NO synthesis, thereby addressing two critical mechanisms in cancer progression [7, 8]. The growing emphasis on botanical remedies underscores the potential of wild plants and their phenolic compounds in developing functional foods and nutraceuticals with significant health-promoting and therapeutic benefits.

Despite extensive research on *Heliotropium*, the pharmacological properties and phenolic composition of *Heliotropium dolosum* De Not., commonly known as “bambulotu” in Türkiye, remain underexplored. This study provides the first comprehensive evaluation of the antioxidant, cytotoxic, and NO-inhibitory activities of *H. dolosum* aerial extracts, alongside its phenolic profile, contributing novel insights into its therapeutic potential.

This study set out to assess the antioxidant, cytotoxic, and NO-inhibitory properties of extracts from *H. dolosum* and to identify the bioactive polyphenolic compounds contributing to these effects.

## Materials and Methods

The material and methods section has been presented as supplementary material.

## Results and Discussion

Numerous peer-reviewed studies on medicinal plants published by research groups demonstrate how deeply active herbal medicine research is in this day and age. Many conventional beliefs have been affirmed, rejected, or re-adopted, and countless novel perspectives have been declared. But even if we acknowledge the volume of research being done, the need to find new plant-based medications remains constant [9].

*Heliotropium* stands out as a significant botanical genus with deep-rooted cultural significance, making it a promising reservoir of bioactive compounds [10]. *Heliotropium* species have long been utilized in various traditional medicinal practices to treat an array of ailments, including rheumatism, gout, and as febrifuges, antiseptics, anti-inflammatory agents, cholagogues, and wound healers [11]. Although no documented ethnobotanical uses of *H. dolosum* exist in the literature, it is known to be employed by Anatolian communities in Türkiye as a remedy for scorpion stings and to promote wound healing.

## Yield of Extract and Total Amount of Secondary Metabolites

Calculations were made to determine the effectiveness of extracts made with solvents of varying polarity. The yield of extractions is listed in Table 1. Water extract yielded the largest amount of extract (22.43%). The fact that water has the highest polarity may be connected to this outcome.

The methanol extract had the highest total phenolic content, which was determined to be equal to gallic acid (37.12 mgGAEs/g). Our results showed that methanol extract has the highest total flavonoid amount (36.30 mgQEs/g), while water extract has the highest total saponin amount (27.19 mgQAEs/g).

**Table 1:** Extract yield and total secondary metabolites amount of *H. Dolosum* according to different solvents (mean ± SD)

	Chloroform	Ethanol	Methanol	Water
Extraction yield (%)	5.06 ± 0.09^a^	10.04 ± 0.15^a^	18.67 ± 0.23^b^	22.43 ± 0.33^b^
TPA (mg GAEs/g)	11.08 ± 0.17^a^	23.35 ± 0.35^b^	37.12 ± 0.48^d^	28.60 ± 0.40^c^
TFA (mg QEs/g)	4.95 ± 0.07^a^	18.46 ± 0.21^b^	36.30 ± 0.41^d^	26.08 ± 0.37^c^
TSA (mg QAEs/g)	Nd	12.27 ± 0.18^a^	17.64 ± 0.20^a^	27.19 ± 0.45^b^

*TPA*: total phenolic amount; *TFA*: total flavonoid amount; *TSA*: total saponin amount; *GAEs*: gallic acid equivalents; *QEs*: quercetin equivalents; *QAEs*: quillaja equivalents, *nd*: not detected. In each row, different letters indicate significant difference (*P* ≤0.05)

Although the *Heliotropium* genera are an important producer of bioactive chemicals [12], to the best of our knowledge, the polyphenolic composition of *H. dolosum* has not been elucidated. However, a previous study reported the total phenolic content of the aqueous extract of *H. dolosum* as 122.7 mg GAE/g dry weight [13]. In addition to the solvent used, factors such as geographical, physiological, and genetic variations can significantly affect the secondary metabolite content of plants [14]. The total phenolic and flavonoid amounts of *H. crispum* methanol extract were reported earlier as 24.84 mg GAE/g and 19.73 mg REs/g, respectively [10]. Based on these findings, *H. dolosum* methanolic extract used in the current study had a higher total phenolic amount (37.12 mg GAEs/g) than *H. crispum.* Our results regarding the occurrence of higher phenolic compounds in methanolic extract are supported by the published outcomes.

## Antioxidant Activity

To determine the antioxidant activity of *H. dolosum* extracts, we favored six diverse methods to compare the results with each other and provide more reliable data (Table 2). β-carotene/linoleic acid and phosphomolybdenum assay were used to evaluate the total antioxidant capacity of the extracts. According to the β-carotene/linoleic acid assay, water extracts (77.81%) possess the strongest antioxidant activity; however, in the phosphomolybdenum assay, methanol extracts (83.74 mgAEs/g) possess the most efficient activity.

Assays for DPPH and ABTS radical scavenging are frequently used to quickly assess antioxidant activity because of their stability in the radical form and simplicity of the assay. The highest radical scavenging activity was demonstrated by methanol extracts in both the DPPH (IC_50_: 26.14 µg/mL) and ABTS (IC_50_: 41.15 µg/mL) tests. To ascertain the extracts’ reduction power, the FRAP assay was used. Results that are comparable to the trolox standard demonstrate the high reducing power activity of aqueous extracts (102.04 mgTEs/g). Additionally, the extracts’ ability to chelate ferrious metals was assessed, and the results were reported as the standard equivalent of EDTA. According to this test, methanolic extract had the highest chelating capacity (35.08 mgTEs/g) (*P* ≤0.05).

**Table 2:** Antioxidant properties of extracts from *H. Dolosum* (mean ± SD)

	Chloroform	Ethanol	Methanol	Water	BHT
β-carotene/linoleic acid assay (% inhibition)	52.07 ± 1.13^a^	64.35 ± 1.38^a^	72.23 ± 1.42^b^	77.81 ± 1.48^b^	94.11 ± 1.68^c^
Phosphomolybdenum assay (mg AEs/g)	60.24 ± 1.35^a^	71.40 ± 1.45^a^	83.74 ± 0.61^b^	81.01 ± 0.52^b^	Nd
DPPH assay (IC_50_ value, µg/mL)	118.03 ± 1.75^d^	32.56 ± 0.65^b^	26.14 ± 0.35^b^	80.01 ± 0.48^c^	9.45 ± 0.07^a^
ABTS assay (IC_50_ value, µg/mL)	127.54 ± 1.81^d^	48.67 ± 1.10^b^	41.15 ± 0.08^b^	74.33 ± 1.45^c^	18.41 ± 0.33^a^
FRAP assay (mg TEs/g)	40.36 ± 0.06^a^	85.22 ± 0.65^b^	95.70 ± 1.70^b^	102.04 ± 1.73^c^	Nd
Metal chelating activity (mg EDTAEs/g)	10.87 ± 1.05^a^	17.66 ± 0.31^a^	35.08 ± 0.68^b^	28.57 ± 0.34^b^	Nd

*AEs*: ascorbic acid equivalents, *TEs*: trolox equivalents, *EDTAEs*: EDTA equivalents, *nd*: not detected. In each row, different letters indicate significant difference (*P* ≤0.05)

**Fig. 1:**
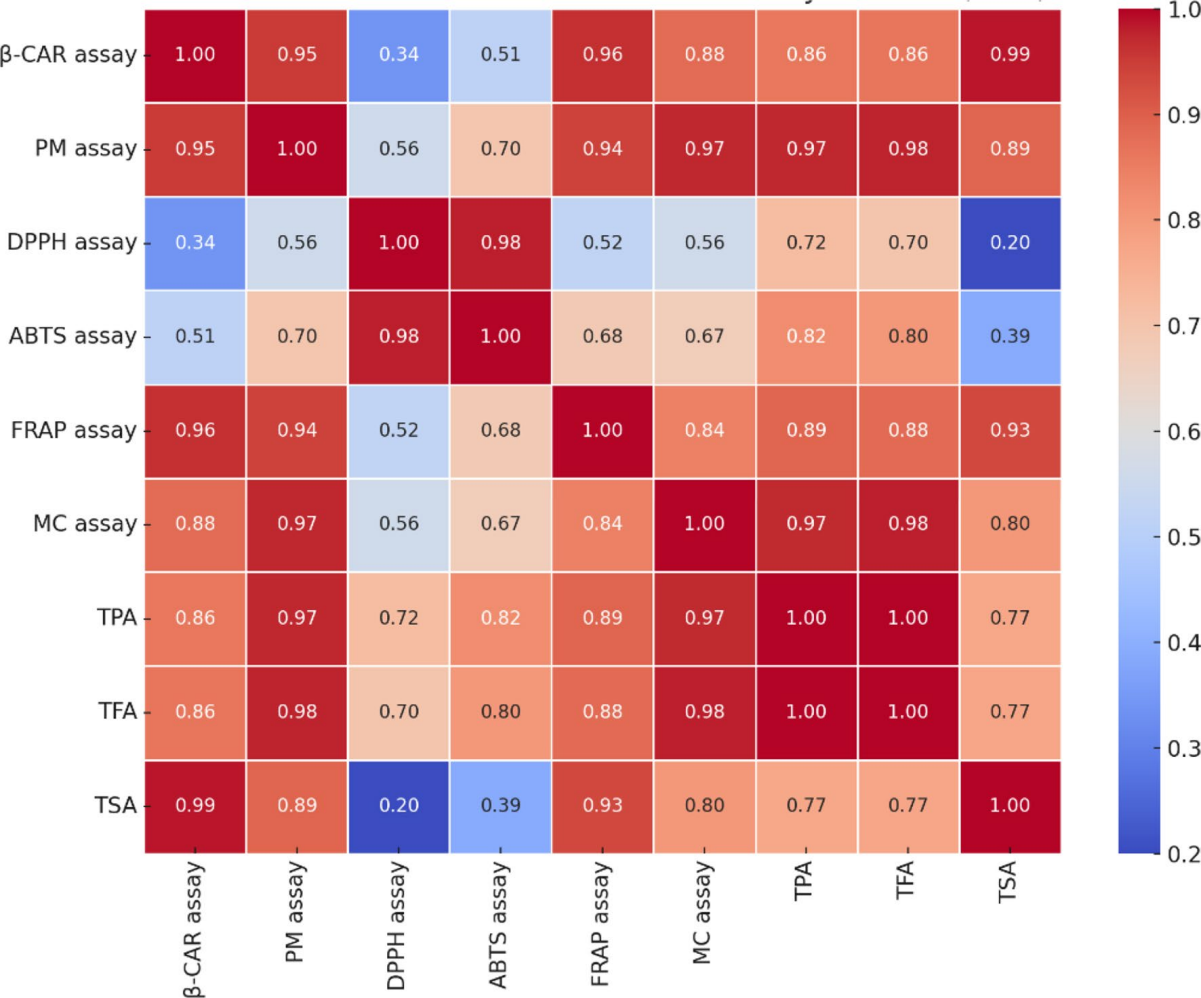
Pearson correlation between antioxidant tests and total secondary metabolite contents (higher correlation coefficients (red colour) indicate a stronger positive relationship, while lower correlation coefficients (blue colour) indicate weaker relationships)

Antioxidants neutralize reactive oxygen species, preventing their harmful effects and reducing the risk of disorders such as cardiovascular diseases, cancer, and diabetes [15]. Since no single method can fully evaluate the antioxidant potential of plant extracts due to the complexity of bioactive molecules, six different assays were employed to assess the antioxidant activity of *H. dolosum* extracts. The correlation analyses between antioxidant activity tests and the total amount of secondary metabolites were conducted using the Pearson correlation test (Fig. 1). The correlation coefficients between antioxidant activity tests and TPA and TFA were generally found to be high. Strong positive correlations were observed between polyphenolic contents and antioxidant activities. Also, the methanolic extracts showed the highest levels of total phenolics, flavonoids, and antioxidant capacity, including radical scavenging, metal-reducing, and chelating activities. These results, consistent with previous studies, highlight a strong correlation between phenolic content and antioxidant activity [15, 16].

## Brine Shrimp Lethality Test (BSLT)

The potential toxicity of ethanol, methanol, chloroform and water extracts of *H. dolosum* (10–1000 µg/mL) was evaluated on brine shrimp lethality assay using etoposide a standard drug presented in Table 3. BSLT results are expressed as LC_50_ (concentration to kill 50% of the brine shrimp). With an LC_50_ value of 18.1 µg/mL, the methanolic extract demonstrated significant cytotoxicity, indicating that it is more toxic compared to the other extracts and accepted as bioactive due to lower LC_50_. As the concentration of each extract increased, it also raised the mortality rate.

**Table 3:** BSLT assay results of *H. Dolosum* extracts on brine shrimp nauplii^a^

Tested extracts (10–1000 µg mL^− 1^)	LC_50_ (µg mL^− 1^)	Confidence intervals 95%
Chloroform	67.4	32.1–97.5
Ethanol	23.0	8.4–52.2
Methanol	18.1	10.7–27.3
Water	25.7	9.8–58.6

^a^Standard drug; etoposide, LC_50_ = 7.52 µg mL^− 1^

For development of anticancer drugs, cytotoxic activity is preliminary screening. Recently, wide number of plants have been examined for their antitumor properties. To ascertain the cytotoxic effect of *H. dolosum* extracts, we chose BSLT. Less than 1000 µg/mL is considered bioactive (toxic) based on LC_50_ values when using BSLT to determine the toxicity of plant extracts [17]. The order of toxicity for brine shrimps was methanol > ethanol > water > chloroform. The cytotoxic effect of methanol extract was 18.1 µg/mL (< 1000 µg/mL). It suggests that the methanol extract may contain a higher concentration of plant components with cytotoxic ability. Since brine shrimp toxicity is positively correlated with certain human cancers that are solid and nasopharyngeal carcinoma, it can be concluded that *H. dolosum* has a cytotoxic effect and could therefore be a source of anticancer compounds [18]. Since the methanolic extract exhibited high activity in both antioxidant activity and BSLT, further biological activity assays were conducted using methanol as the solvent.

## Cytotoxic Activity

**Fig. 2:**
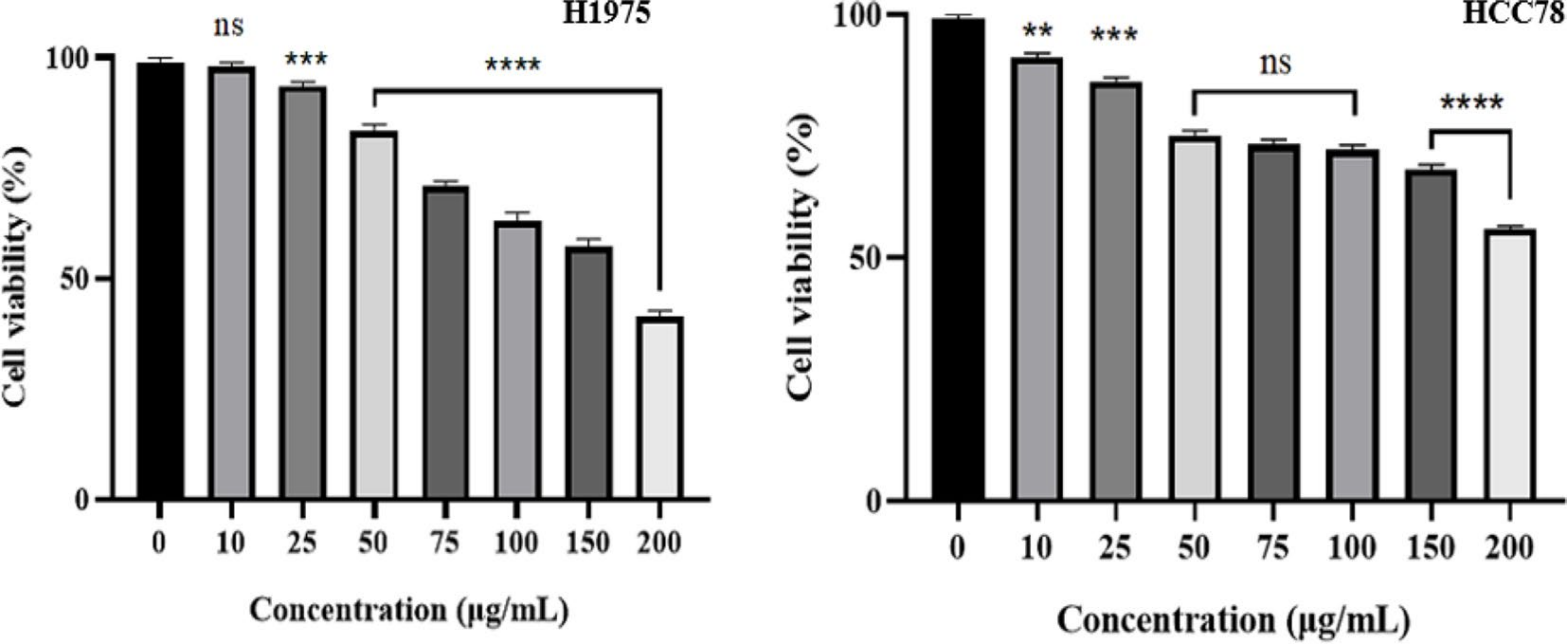
Concentration-dependent inhibitory effect of *H.dolosum* methanol extract on viability of cancer cell line H1975 and HCC78 cells. Data are presented as mean ± SD. *P ≤0.05, **P ≤0.01, ***P ≤0.001, ****P ≤0.0001, ns - no significance (*P >* 0.05)

The effect of *H. dolosum* methanol extract (10–200 µg/mL) on cell viability of NSCLC cells was determined by using CellTiter Glo assay. There was a dose-dependent decrease in survival for both H1975 and HCC78 cells (*P* ≤0.05) (Fig. 2). The IC_50_ values (µg/mL) that cause 50% cell death were calculated at 151.34 µg/mL and 197.06 µg/mL on H1975 and HCC78 cells, respectively.

Lung cancer is one of the fatal cancers for humankind. NSCLC accounts for the majority of (85%) whole lung cancers. Products derived from plants exhibit promising sources of anti cancer compounds with fewer side effects than synthetic drugs [19]. In a concentration-dependent manner (*P* ≤0.05), *H. dolosum* methanol extract decreased the cell viability of H1975 and HCC78 cells. This cytotoxic activity may be caused by rutin and epicatechin, which are the major flavonoids of *H. dolosum*. These flavonoids have been proven as anticancer agents in the literature [20, 21]. In a previous study, Fayed et al. [11] evaluated the cytotoxic potential of *Heliotropium ramosissimum* methanolic extract against six cancer cell lines, including human melanoma (A-375), colorectal cancer (Colo-205), hepatocellular carcinoma (HepG-2), cervical cancer (HeLa), and large cell lung cancer (H-460). The study demonstrated that the methanolic extract exhibited significant cytotoxicity across all tested cell lines, with particularly strong activity against the Colo-205 colorectal cancer cell line (IC_50_ = 18.60 µg/mL). The authors attributed this activity to the presence of phenolic compounds, such as isochlorogenic acid and orientin, which likely contribute to apoptosis induction and the reduction of cancer cell viability.

### Nitric Oxide Inhibitory Activity

**Fig. 3:**
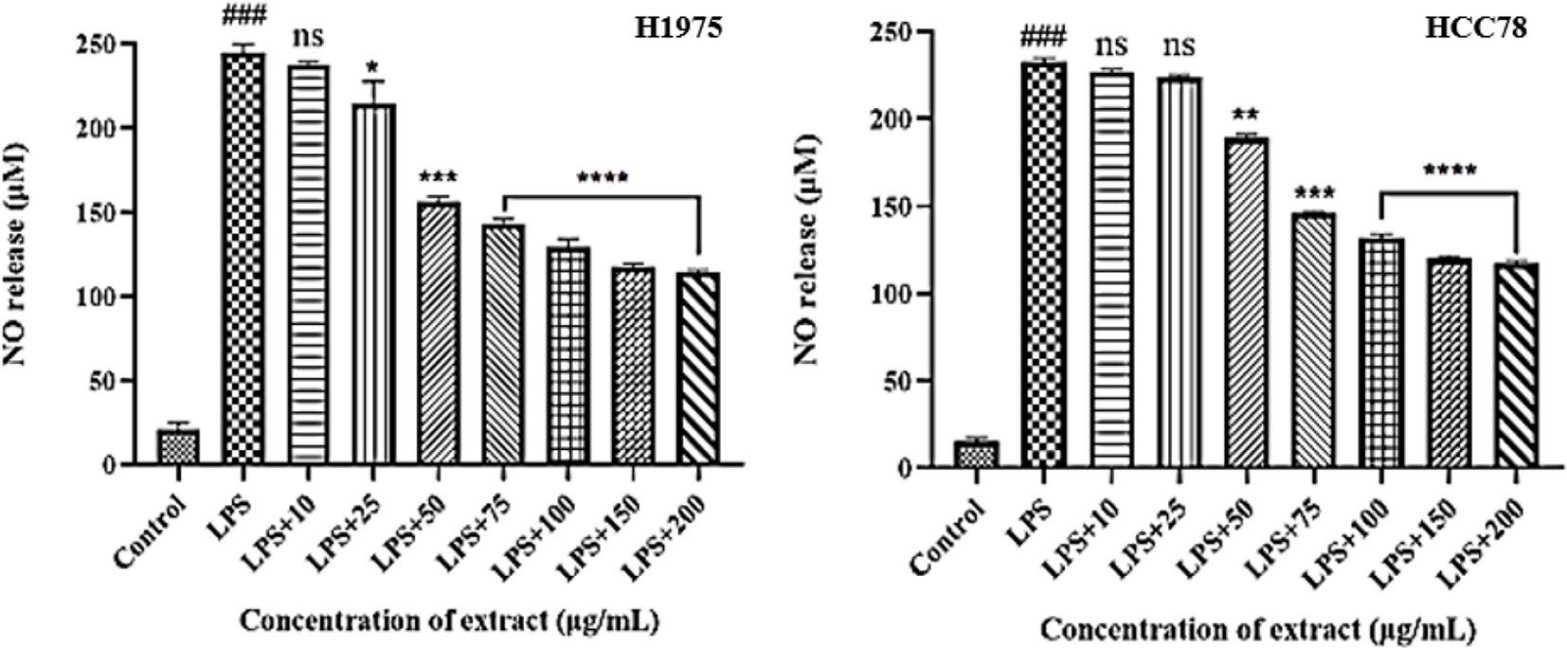
The effect of *H.dolosum* methanol extract on NO production of cancer cell line H1975 and HCC78 cells. Data are presented as mean ± SD. ^#^*P*≤0.05, ^##^*P*≤0.01, ^###^*P*≤0.001 compared with control group; *P ≤0.05, **P ≤0.01, ***P ≤0.001, ****P ≤0.0001 compared with LPS group, ns - no significance (*P >* 0.05)

The inhibitory effect of *H. dolosum* methanol extract (10–200 µg/mL) on LPS-activated NSCLC cells was measured by using Griess reaction, as an index of NO release. As a measure of NO production, the amount of nitrite in the growth medium was determined. The amount of nitrite (µM), a stable metabolite of NO, diminished as a result of the increasing extract concentration (*P* ≤0.05). H1975 and HCC78 cells’ nitrite levels varied from 20.67 to 114.33 and 15.56 to 117.75 µM, respectively. NO inhibitory activity of *H. dolosum* extract in LPS-stimulated cells is demonstrated in Fig. 3.

As an effector molecule, NO, a reactive nitrogen species, has a variety of functions in various biological systems, such as antimicrobial, antitumor, and neuronal communication [22]. Despite the fact that high NO concentrations are cytotoxic, the amounts generated in a large number of human cancers may promote tumor growth and spread [23]. Clinical observations have shown that NO levels in the lungs of lung cancer patients were raised in compared to those of normal subjects [24]. Numerous in vivo and in vitro investigations have indicated that NOS inhibitors inhibit the development of cancer. In this context, selective inhibitors of NOS may have a curative task in specific cancers [25]. In this study, we detected that *H. dolosum* prevented NO formation in LPS-activated H1975 and HCC78 cells. These findings suggest significant contribution to acquire a novel bioactive compound with anticancer activity from *H. dolosum*.

## Phenolic Compound Characterization by HPLC

Using 15 phenolic compounds as standards, the HPLC method was used to determine the phenolic components in the methanol extracts of the aerial part of *H. dolosum*. Table 4 presents the different amounts of phenolic components that were found in the extract.

Based on these findings, caffeic acid (12307.19 µg/g), 2,5-dihydroxybenzoic acid (5710.19 µg/g), ellagic acid (5061.65 µg/g), rutin (2487.37 µg/g) and epicatechin (1969.35 µg/g) are the most abundant phenolic components found in *H. dolosum* methanol extract.

**Table 4:** HPLC-based characterization of phenolic components in methanolic *H. dolosum* extract (mean ± SD)

No	Identified phenolic Compounds	RT (min)	UV_max_ (nm)	LOD (µg/mL)	µg/g extract (mean ± SD)
1	Gallic acid	6.8	280	0.015	57.96 ± 0.55
2	3,4-dihydroxybenzoic acid	10.7	280	0.031	52.65 ± 0.49
3	4-hydroxybenzoic acid	15.7	280	0.014	44.44 ± 0.41
4	2,5-dihydroxybenzoic acid	17.2	320	0.753	5710.19 ± 118.1
5	Chlorogenic acid	18.2	320	0.011	856.46 ± 7.28
6	Vanillic acid	19.2	320	0.112	57.12 ± 0.50
7	Epicatechin	21.3	260	0.433	1969.35 ± 40.12
8	Caffeic acid	22.7	320	0.018	12307.19 ± 240.1
9	*p*-coumaric acid	26.1	320	0.020	298.77 ± 3.02
10	Ferulic acid	30.1	320	0.012	99.75 ± 1.09
11	Rutin	45.6	360	0.576	2487.37 ± 47.21
12	Ellagic acid	47.7	240	0.455	5061.65 ± 96.33
13	Naringin	49.7	280	0.404	803.03 ± 5.01
14	Cinnamic acid	67.8	280	0.016	145.64 ± 1.77
15	Quercetin	71.1	360	0.578	64.15 ± 0.58

RT: retention time, LOD: limit of detection

While extensive research has been conducted on the alkaloid profiles of the *Heliotropium* genus, studies focusing on its phenolic constituents remain relatively scarce [10, 11]. To date, the phenolic compounds identified from various *Heliotropium* species are presented in Table 5.

**Table 5:** Composition of the phenolic constituents of other *Heliotropium* species in the literatüre: a focus on the most abundant phenolic acids and flavonoids

Species	Plant parts	Extraction solvents	Major phenolic constituents		References
			Phenolic acids	Flavonoids	
*Heliotropium crispum* Desf.	Whole plant	Methanol	Ferulic acid Lithospermic acid Salvianolic acid A *p*-salicylic acid	Cimifugin Rivenprost Tamadone Wightin	[10]
*Heliotropium curassavicum* L.	Aerial parts	Methanol	Chlorogenic acid Ellagic acid Gallic acid Syringic acid	Catechin Naringenin Rutin Quercetin	[26]
*Heliotropium europaeum* L.	Aerial parts	Methanol	Caffeic acid 2,5-dihydroxybenzoic acid	Epicatechin Quercetin	[16]
*Heliotropium indicum* L.	Leaves	Methanol	Chlorogenic acid Syringic acid Sinapic acid	Catechin hydrate	[27]
*Heliotropium indicum* L.	Whole plant	Ethyl acetate	Caffeic acid Gallic acid Tannic acid	Quercetin Naringenin	[28]
*Heliotropium procumbens* Mill.	Aerial parts	Methanol	Dihydroxybenzoic acid Ferulic acid Caffeic acid Rosmarinic acid Salvianolic acid B Lithospermic acid Syringic acid	Kaempferol Luteolin-7-O-glucoside Naringenin-7-O-glucoside	[29]
*Heliotropium ramosissimum* (Lehm.) DC.	Aerial parts	Methanol	Caffeic acid, Cinnamic acid Protocatechuic acid 4-hydroxybenzoic acid	Epicatechin Eriodictyol Kaempferol Quercetin	[11]
*Heliotropium dolosum* De Not.	Aerial parts	Methanol	Caffeic acid Chlorogenic acid Ellagic acid 2,5-dihydroxybenzoic acid	Epicatechin Naringin Rutin	This study

Because of methanol extract has a higher biological activities and total secondary metabolites amount than others, methanol extract was used in HPLC analysis to elucidate the polyphenolic profile of *H. dolosum*. While the major phenolic acids in the extract were caffeic acid, 2,5-dihydroxybenzoic acid, ellagic acid and chlorogenic acid, the main flavonoids were rutin, epicatechin and naringin. Other compounds found in lower concentrations were 3,4-dihydroxybenzoic acid, gallic acid, 4-hydroxybenzoic acid, vanillic acid, *p*-coumaric acid, ferulic acid, cinnamic acid and quercetin.

Polyphenols, key bioactive components in plants and natural extracts, are known for their therapeutic effects. In the Boraginaceae family, polyphenols and flavonoids exhibit diverse pharmaceutical activities, including antioxidant, antiviral, antibacterial, anti-inflammatory, and hepatoprotective effects [30]. *H. dolosum* contains abundant phenolic acids, such as caffeic acid, gentisic acid, ellagic acid, and chlorogenic acid, which are documented for their free radical scavenging and metal-chelating activities [31]. The observed antioxidant activities in polar extracts may be attributed to their phenolic acid content, as supported by literature and test results.

## Conclusion

In conclusion, the methanol extract of *H. dolosum* exhibited cytotoxic potential, multiple individual polyphenolic compounds with antioxidant tendencies, and consistently high total phenolic and flavonoid contents. *H. dolosum*’s notable toxicity to brine shrimp, NO inhibitory activity in LPS-stimulated cancer cells, and antioxidant activities may open the door to more research into this plant in order to isolate the pure compounds that give rise to the biological characteristics that have been observed.

## Electronic Supplementary Material

Below is the link to the electronic supplementary material.

Supplementary Material 1

## Data Availability

No datasets were generated or analysed during the current study.
