# Supplementary material for: Unveiling the Cytotoxic and NO Inhibitory Potential of Heliotropium dolosum Extracts from Türkiye: A First Insight Into Its Phenolic Profile
Source: Plant Foods Hum Nutr. 2025 Mar 6;80(1):86. doi: 10.1007/s11130-025-01313-y (PMC11885333; doi:10.1007/s11130-025-01313-y)
Supplement: Supplementary file 1 — Supplementary Material 1 [file 11130_2025_1313_MOESM1_ESM.docx]

**Unveiling the Cytotoxic and NO Inhibitory Potential of Heliotropium dolosum Extracts from Türkiye: A First Insight into Its Phenolic Profile**

**Cennet Özay***

*Department of Basic Pharmaceutical Sciences, Faculty of Pharmacy, Izmir Katip Celebi University, 35620 Izmir, Türkiye.

**Address for correspondence:** Assist. Prof. Cennet OZAY, Ph.D. Department of Basic Pharmaceutical Sciences, Faculty of Pharmacy, Izmir Katip Celebi University, 35620 Izmir, Türkiye. E-mail: cennet.ozay@ikcu.edu.tr, ORCID ID: 0000-0002-1120-6122

**Materials and Methods**

The material and methods section has been presented as supplementary material.

**Plant Material and Extraction**

In October 2013, the *H. dolosum* individuals were gathered from Denizli, Turkey and identified in our laboratory (Voucher No: C. Ozay 3001). In a shaker water bath set at 48-50°C for six hours, ethanol, methanol, chloroform, and water were used to prepare the extracts. These extracts were filtered and the solvents were removed in vacuum by a rotary evaporator at 45-50°C and then lyophilized. Until they were needed, the crude extracts were stored at -20°C. Weighing the dried extracts allowed us to calculate the yield percentage [1]. This formula, W1/W0 × 100, was used to determine the yield percentage. where W0 is the sample's initial weight and W1 is the extract's final weight.

**The Calculation of the Total Amount of Secondary Metabolites**

As stated in the previous paper [2], colorimetric assays were employed to determine the extracts' total levels of phenolic, flavonoid, and saponin contents. Gallic acid (mg GAEs/g), quercetin (mg QEs/g), and quillaja (mg QAEs/g) equivalents were used to represent these contents, respectively. All the assays were performed in triplicate and the results were reported as mean ± standard deviation (SD).

**HPLC Characterization of Phenolic Compounds**

RP-HPLC was utilized to examine the phenolic chemicals found in *H. dolosum*. **The dried crude extract was dissolved in methanol at a concentration of 1 mg/mL for RP-HPLC analysis.** Using a reversed-phase column and a mobile phase consisting of a mixture of two solvents (methanol and 3% acetic acid), separation was carried out at 30°C. The mobile phase flowed at a rate of 0.8 mL/min. A diode array detector was used to measure the phenolic acids at 280 nm and the flavones and flavonols at 320 and 360 nm. The phenolic compounds in the methanolic extract of H. dolosum were quantified as μg/g extract, with each target compound identified using a combination of spectrum matching and retention time, and their concentrations determined based on peak areas relative to calibration curves established from standard compounds.

### **Antioxidant Activity Tests**

The antioxidant potential of the extracts was assessed through a combination of methods, each highlighting different aspects of its activity. The DPPH radical scavenging assay involved incubating the extracts with DPPH methanolic solution for half an hour without light, then a measurement of absorbance at 517 nm, with results expressed as IC_50_ values and BHT serving as the positive control. Similarly, the ABTS radical cation assay, based on a modified method by Re et al. measured the interaction between the extracts and the ABTS solution at 734 nm after half an hour, also reporting IC_50_ values with BHT as the reference [3].

The β-carotene/linoleic acid assay was used to assess the prevention of linoleic acid oxidation, calculating inhibition percentages using a β-carotene system as described by Ozay and Mammadov [1]. Furthermore, Mo(VI) was reduced to Mo(V) by the phosphomolybdenum (PM) assay, which measured the green hue at 695 nm. According to Prieto et al. [4], the results were presented as the equivalent of ascorbic acid (mg AEs/g extract).

Fe(III)-TPTZ was reduced to Fe(II)-TPTZ at 593 nm using the ferric reducing antioxidant power (FRAP) assay, presenting results as trolox equivalents (mg TEs/g extract) based on Apak et al. [5]. Finally, the metal chelating activity of the extracts, determined using a modified Dinis et al. [6] method, assessed their ability to inhibit Fe^2+^-ferrozine complex formation, with the color intensity measured at 562 nm and results expressed as EDTA equivalents (mg EDTAEs/g extract). All the antioxidant activity tests were conducted in triplicates.

**Brine Shrimp (*Artemia salina* L.) Lethality Test**

The brine shrimp lethality test (BSLT) was performed as previously reported [1]. Briefly, for 48 hours, *A. salina* eggs were left to develop at 28 °C in saltwater to reach the mature larvae stage called nauplii and then all nauplii were bred for 24h in the presence of *H. dolosum* extracts (10-1000 μg/mL) dissolved in the brine solution. After the incubation period (24h) with extracts, the number of surviving shrimps was evaluated and their vitality was compared to the untreated control group. Cytotoxic drug, etoposide was used as a positive control. Data analysis was achieved by EPA Probit Analysis program to determine the LC_50_ values. The BSLT assay for each extract was conducted in triplicate.

**Cytotoxicity Assay**

Human non-small cell lung cancer (NSCLC) cells H1975 and HCC78 were cultivated in RPMI 1640 medium in a CO_2_ incubator at 37 °C. In short, 96-well plates were seeded with 2×10^3^ cells per well. Cells were exposed to extracts at varying concentrations (10-200 μg/mL) for 24 hours.

For the control group, cells were not treated with any extract. At the end of the incubation time, cell viability was assessed by using a CellTiter-Glo^®^ mixture as recommended by the supplier. ATP-based luminometric measurement from the metabolically active cells in the culture was determined by CellTiter-Glo^®^ luminescent cell viability assay and luminescence was measured on the GloMax®-Multi Detection System (Promega). The percentage of cell viability was calculated relative to control cells. The cytotoxicity assays were conducted in triplicates.

**Nitric Oxide Assay**

The NO assay was carried out, yet a little differently than previously described [7]. After preincubation of H1975 and HCC78 cells (2×10^3^ cells/well) with lipopolysaccharide (LPS, 1μg/mL, 24h) for NO production, the extracts (10-200 μg/mL) were added and incubated for 48h. Cells in the untreated control group received neither LPS nor any extract treatment. An indicator of NO production was the amount of nitrite in the culture medium. The amount of nitrite (µM), a stable NO product, was measured with the Griess reagent (1% sulfanilamide and

0.1% naphthylethylenediamine dihydrochloride in 2.5% phosphoric acid). Briefly, 100 μL of cell culture medium was mixed with 100 μL of Griess reagent. Afterwards, the mixture was incubated for 10 min at room temperature and the absorbance of the chromophore that formed during diazotization of the nitrite with sulfanilamide and subsequent coupling with naphthylethylenediamine dihydrochloride was immediately read at 560nm using a microplate reader. The nitric oxide assay was performed in triplicate.

**Statistics**

GraphPad 8.0 was used for the statistical analyses. One-way ANOVA and Tukey's test were used for statistical comparisons. *P* ≤0.05 denotes significance. The correlation analyses between antioxidant activity tests and the total amount of secondary metabolites were conducted using the Pearson correlation test.

**References**

1. Ozay C, Mammadov R (2022) Phenolic profile, antioxidant, and antiproliferative activities of *Convolvulus aucheri* Choisy. Vojnosanit Pregl 79:488-495. https://doi.org/10.2298/VSP200114133O

2. Ozay C, Mammadov R (2019) Antioxidant activity, total phenolic, flavonoid and saponin contents of different solvent extracts of *Convolvulus phrygius* Bornm. Curr Pers MAPs 2:23-28. https://doi.org/10.38093/cupmap.567809

3. Re R, Pellegrini N, Proteggente A et al (1999) Antioxidant activity applying an improved ABTS radical cation decolorization assay. Free Radic. Biol. Med 26:1231-1237. https://doi.org/10.1016/S0891-5849(98)00315-3

4. Prieto P, Pineda M, Aguilar M (1999) Spectrophotometric quantitation of antioxidant capacity through the formation of a phosphomolybdenum complex: specific application to the determination of vitamin E. Anal Biochem 269:337-341. https://doi.org/10.1006/abio.1999.4019

5. Apak R, Özyürek M, Güçlü K et al (2016) Antioxidant Activity/Capacity Measurement. 1. Classification, Physicochemical Principles, Mechanisms, and Electron Transfer (ET)-Based Assays. J Agric Food Chem 64:997-1027. https://doi.org/10.1021/acs.jafc.5b04739

6. Dinis TC, Maderia VM, Almeida LM (1994). Action of phenolic derivatives (acetaminophen, salicylate, and 5-aminosalicylate) as inhibitors of membrane lipid peroxidation and as peroxyl radical scavengers. Arch Biochem Biophys 315:161-169. https://doi.org/10.1006/abbi.1994.1485

7. Yang EJ, Yim EY, Song G et al (2009) Inhibition of nitric oxide production in lipopolysaccharide-activated RAW 264.7 macrophages by Jeju plant extracts. Interdiscip Toxicol 2:245-249. https://doi.org/10.2478/v10102-009-0022-2
